# Integrated web service for improving alignment quality based on segments comparison

**DOI:** 10.1186/1471-2105-5-98

**Published:** 2004-07-22

**Authors:** Dariusz Plewczynski, Leszek Rychlewski, Yuzhen Ye, Lukasz Jaroszewski, Adam Godzik

**Affiliations:** 1 Bioinformatics Laboratory, BioInfoBank Institute, Poznan, Poland; 2 Interdisciplinary Centre for Mathematical and Computational Modeling, University of Warsaw, Poland; 3 The Burnham Institute, La Jolla, USA; 4 Bioinformatics Core JCSG, University of California San Diego, La Jolla, USA

**Keywords:** Library of protein motifs, Profile-profile sequence similarity (BLAST, FFAS), Fragments library (FRAGlib), Predicted Local Structure Segments (PLSSs), Segment Alignment (SEA), Network matching problem

## Abstract

**Background:**

Defining blocks forming the global protein structure on the basis of local structural regularity is a very fruitful idea, extensively used in description, and prediction of structure from only sequence information. Over many years the secondary structure elements were used as available building blocks with great success. Specially prepared sets of possible structural motifs can be used to describe similarity between very distant, non-homologous proteins. The reason for utilizing the structural information in the description of proteins is straightforward. Structural comparison is able to detect approximately twice as many distant relationships as sequence comparison at the same error rate.

**Results:**

Here we provide a new fragment library for Local Structure Segment (LSS) prediction called FRAGlib which is integrated with a previously described segment alignment algorithm SEA. A joined FRAGlib/SEA server provides easy access to both algorithms, allowing a one stop alignment service using a novel approach to protein sequence alignment based on a network matching approach. The FRAGlib used as secondary structure prediction achieves only 73% accuracy in Q3 measure, but when combined with the SEA alignment, it achieves a significant improvement in pairwise sequence alignment quality, as compared to previous SEA implementation and other public alignment algorithms. The FRAGlib algorithm takes ~2 min. to search over FRAGlib database for a typical query protein with 500 residues. The SEA service align two typical proteins within circa ~5 min. All supplementary materials (detailed results of all the benchmarks, the list of test proteins and the whole fragments library) are available for download on-line at .

**Conclusions:**

The joined FRAGlib/SEA server will be a valuable tool both for molecular biologists working on protein sequence analysis and for bioinformaticians developing computational methods of structure prediction and alignment of proteins.

## Background

Protein structure is obviously modular, with similar structural segments, such as alpha helices and beta strands found in unrelated proteins. Such segments, identified from structure, are used extensively in description and analysis of protein structures [1,2]. Several groups have demonstrated that only a small library of segments is sufficient to rebuild experimental protein structures with high accuracy [3]. Predicted local structure segments (PLSS) are also used in structural prediction, starting from the nearest neighbor approach to secondary structure prediction [4-6]. This idea was later extended and lead to even more successful applications of PLSSs in *ab initio *structure prediction by Baker and colleagues, who developed a library of sequence-structure motifs called I-sites [7]. Those motifs are later assembled in a complete protein structure by a program ROSETTA [8]. Predicted local structure segments are also used in a novel protein alignment algorithm, based on the comparison of PLSSs for two proteins treated as networks and finding a common path through networks describing the two proteins [9]. The underlying idea in all those approaches is that because global folding constraints can override local preferences, the prediction of structure segments from local sequence is by necessity uncertain. Therefore, instead of trying to predict a correct local structure, all possible local solutions are identified and other constraints (folded structure in Rosetta, or compatible alignment in SEA) are used to identify a globally consistent solution.

Prediction of local structure segments can be approached in two different ways. A first possibility, used in most nearest neighbor secondary structure algorithms, is to use a representative set of proteins with known structure as source of structure segments, but without any restrictions on a number or type of segments. In this approach, we don't make any assumptions about the compositions and distributions of segments in the library and this approach can be compared to unsupervised learning approach. In a second approach, used for instance in the I-site method, only segments from a specifically constructed fragment library are used in prediction, thus this approach is similar to supervised learning. Interestingly, some limited tests suggest that the former approach leads to lower prediction accuracy [10]. The same tests suggested the possibility that different segment libraries could lead to different prediction, and likely, some segment libraries would be better suited to some tasks.

Following this observation, we have developed the FRAGlib – a fragment library specifically designed to complement a segment alignment SEA. SEA alignment algorithm was developed previously in our group [9] and originally used in conjunction with the I-site library. I-site library [7] was originally developed to be used in *ab initio *folding predictions and anecdotal evidence suggested that it may not be ideally suited for alignment purposes. In this note we describe a combined FRAGlib/SEA server and first benchmarking results of this method.

## Implementation

### Database of Short Fragments

FRAGlib is based on the idea of developing a uniform coverage of all known types of local structural regularity with the distribution based on that observed in natural proteins. The collection of segments is constructed using representative set of proteins from the ASTRAL database [11,12]. For each protein in this set, each continuous segment with regular secondary structure, including the flanking residues on both sides, is added to the FRAGlib (see below for details). We do not utilize any further clustering algorithm so our database contains no-unique entries and it is redundant both in terms of structure and sequence information.

Local structure is described by the SLSR (Symbolized Local Stuctures Representation) codes consisting of 11 symbols {*HGEeBdbLlxc*}, each representing a certain backbone dihedral (phi and psi) region [7,13]. Protein local structure is described as a string of local-structure symbols and a local structure segment is defined as a 5–17 amino acid fragment with constant local structural codes. Segments are then extended by two additional residues offset at the beginning, and at the end of a segment. We store all such segments with their sequence, SLSR style local structures representation codes and the homology profile [14,15], derived from that of their parent protein. The library is highly redundant, i.e. there are many segments with the same structural description, but each of the redundant fragments is coming from a different parent protein (or a different part of the same parent protein), therefore it has a different sequence and a different profile associated with it.

### FRAGlib prediction

In a next step, FRAGlib segment library is used to assign local structure segments for a new protein (query) based only on sequence information using a variant of the FFAS profile-profile alignment algorithm [16]. A profile for the query protein is calculated following the FFAS protocol, then for all possible overlapping segments of length from 7 to 19 amino acids, their profiles are compared to those of the segments from the FRAGlib database and the score of each alignment is calculated using a FFAS-like scalar product of composition vectors at each position. Since the segments being compared have the same length, no dynamic programming alignment is necessary and the score calculation can be highly optimized.

**Figure 1:**
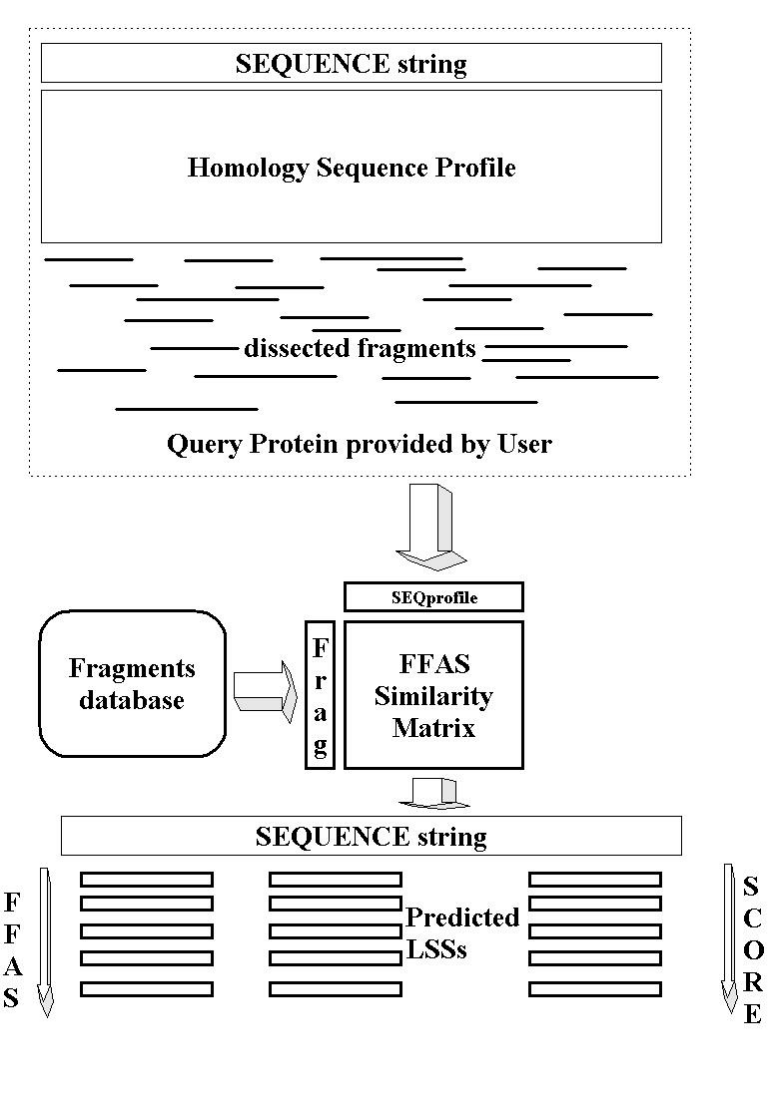
The FRAGlib fragments database is build from ASTRAL representative subset of SCOP database using 40% sequence similarity threshold (see right picture). We store the symbolized local structure representation codes of each fragment together with the homology sequence profile (see left picture). Both are dissected from the SLSR codes and homology profile of a parent protein. The string of SLSR codes representing the local structure of the Cα chain in the phi-phi space. We remove from the fragments database all identical in terms of both SLSR codes and sequence homology profile fragments. On the left picture we present the FRAGlib module for prediction of local structural segments using homology profile similarity and the fragments database. The Query protein is dissected into short parts (from 7 up to 19 residues long). For each part the similarity search is performed. Any member of the fragments database which is similar in terms of homology sequence profile similarity is added to the list of predicted structures for this short part of query protein. This list is then sorted and cut after arbitrary chosen 20^th ^position. If the highest score of predicted fragments is below the user's cut-off value whole prediction is discarder. In the end some of parts of a query protein are covered by list of 20 fragments from the database. They are called the predicted local structural segments (PLSSs).

As the result of this procedure, each position in the query protein can be assigned to all of the possible LSSs in the database, each with a specific score (see Figure 1). Only reduced sets of predicted LSSs, rather arbitrarily limited to the first 20 highest scoring segments are kept for further analysis. This cut-off is the only free parameter of the method, and can be set by user using the Web interface of the server. The Q3 quality of the FRAGlib used as a secondary structure prediction algorithm (data not shown), with the prediction based on the single best scoring segment for each position is 73% on a standard secondary structure prediction benchmark. The Q3 gives percentage of residues predicted correctly as helix, strand, and coil or for all three conformational states.

### SEA Segment Alignment Approach to Protein Comparison

The principal motivation to develop the FRAGlib segment prediction was to further improve the alignment quality for comparing distantly related proteins, which is one of the most important problems in practical application of comparative modeling and fold recognition [17]. To address this problem, we have previously developed a SEA algorithm, which compares the network of predicted local structure segments (PLSSs) for two proteins using the network matching approach. In a previous paper we have demonstrated that the SEA algorithm, using I-site server for PLSSs prediction and a simple sequence-sequence scoring for segment comparison resulted in alignments better than the FFAS profile-profile alignment algorithm and several other alignment tools.

A full description of the SEA algorithm is available in the previous manuscript [9], so only a brief summary is presented here. Every residue in each of the proteins being aligned is described as a vertex in the graph. Two artificial vertices are added to the very beginning of each protein as a source vertex, and also at the end as a sink vertex. For each PLSS is described as an edge between the vertices representing its first and last positions. For some PLSS protocols, some parts of the protein may not be covered by any predicted segments, so virtual edges are added to all neighbor residues to form a complete, continuous network. Each assembly of connected PLSSs corresponds to a path in this network. In a next step, PLSSs networks of two proteins are compared by the SEA algorithm. For each pair of positions *i *and *j*, with position *i *coming form the first protein and position *j *from the second protein, all possible segments covering each of the positions must be considered in a combinatorial way and compared to get the optimal similarity score. It is not the sequences or secondary structures at two positions that are compared, but all segments that cover these two positions. This is the main feature of SEA that makes it different from standard sequence pair-wise alignments. The computational complexity of SEA is about *O*(*NMC*1*C*2), where *C*1 and *C*2 are the average numbers of segments that cover a position in each protein (the segment coverage). Detailed description of the SEA mathematical algorithm together with benchmarks results obtained using the I-site server calculated PLSSs network can be found elsewhere [9].

**Figure 2:**
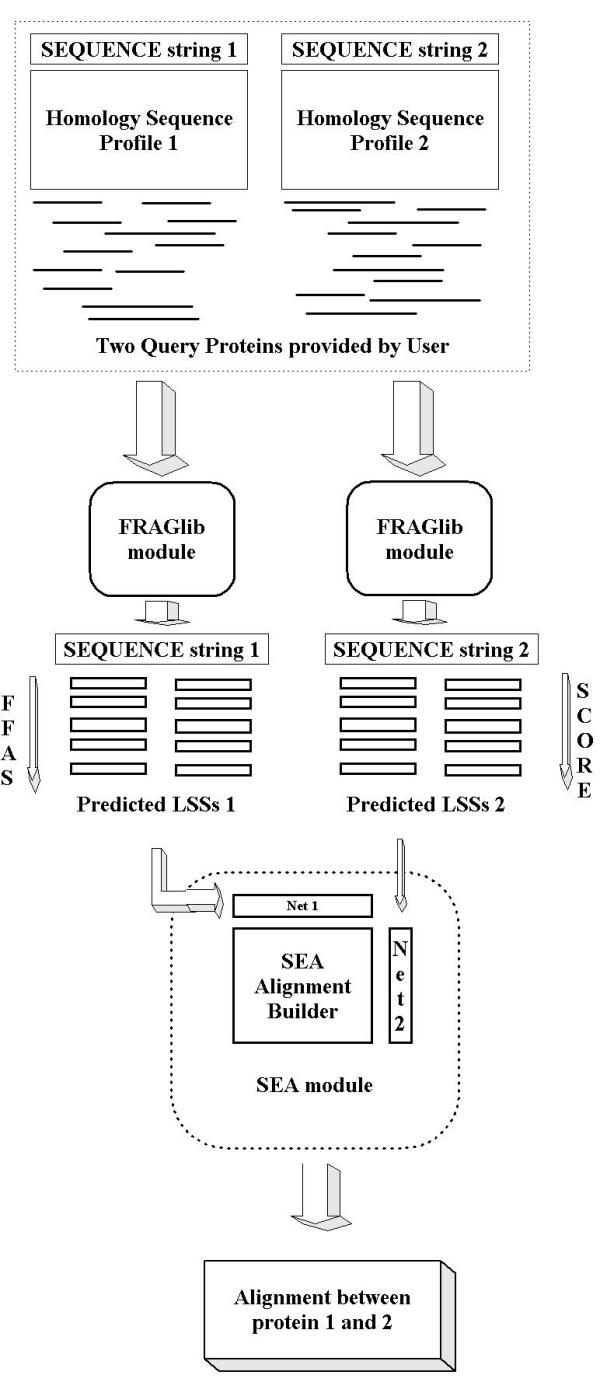
We present here the flowchart of SEA/FRAGlib integrated Web service. The server is based on two modules: the FRAGlib prediction of LSSs and the SEA algorithm for building an alignment between two proteins using comparison of two networks of predicted segments for both of them.

The integrated FRAGlib and SEA server is available at [18]. The FRAGlib database and segment prediction provides the PLSSs network for each aligned protein, and the SEA algorithm aligns the two networks. On Figure 2 we present the flowchart of the integrated web service. Preliminary benchmarks for the FRAGlib/SEA server and presented below. A full paper on the FRAGlib algorithm is in preparation.

## Results and Discussion

We use here as a benchmark the database of 409 family-level similar pairs [19]. Each protein pair shares at least one similar domain as identified by SCOP [20]. Segments coming from the proteins of the same SCOP family as the proteins being compared were removed from the FRAGlib calculated PLSSs network. Further analysis of the SEA results also confirmed that the memorization is not a problem here, as all the SEA alignment are build predominantly from segments that are not locally optimal.

To evaluate the improvement we use two measures of alignment quality: the classical root mean square deviation (RMSD) and the shift score [1]. The shift score measures misalignment between a predicted alignment of two proteins and the reference alignment. The shift score measure ranges from -ε(default as -0.2) to 1.0, where 1.0 means an identical alignment. RMSD is dependent on alignment length and the shift score is dependent on the reference alignment, so both measures are less than perfect in comparing alignments. In our case we use as the reference alignment provided by the CE structural method [21]. We chose the CE, which is available as a single file executable for various operating systems, as an example of purely structural alignment tool. It is a method for fast calculation of pairwise structure alignments, which aligns two proteins chains using characteristics of their local geometry as defined by vectors between Cα positions. Heuristics are used there in defining a set of optimal paths joining termed aligned fragment pairs with gaps as needed. The path with the best RMSD is subject to dynamic programming in order to achieve an optimal alignment. For specific families of proteins additional characteristics are used to weight the alignment.

'Table 1 [see Additional file 5]' compared the quality of the FRAGlib/SEA (identified as SEA_F _in the Table) alignment with that of the structural alignment prepared with the CE algorithm [21] and the SEA algorithm used with I-site segment prediction (SEA_I_), SEA algorithm used with the actual (not predicted) local structure segments (SEA_T_), local single predicted structures (SEA_loc_) and few other publicly available alignment tools. All the results other than the FRAGlib/SEA alignments, as well as alignment quality evaluation, were adopted from the original SEA manuscript [9]. The results presented in 'Table 1 [see Additional file 5]' show that SEA_F _significantly improves the alignment quality as compared to all other methods, including SEA_I _(SEA using I-site prediction), bringing it close to (and in the shift based quality measure actually improving on) the SEA algorithm using the actual structure segments.

## Conclusions

The benchmarks show that SEA with FRAGlib (SEA_F_) integrated prediction service better incorporate diversities of local structure predictions over known methods. It produces also more accurate alignments in comparison to SEA_I _(based on the I-site library), or the SEA with single predicted structures (SEA_loc_). Comparing those sequence pairwise alignments we can observe that predicted local structure information seems to improve the alignment qualities. Alignments from SEA using FRAGlib method of describing diversities of local structure prediction have the same quality as alignments using true local structures derived from their known 3D structures SEA_T_.

## Availability and requirements

An integrated SEA/FRAGlib server is available at [18]. Both components can be used separately, SEA alignment with arbitrary PLSSs and FRAGlib for other purposes than segment alignment, but the integrated server provides the complete alignment method for comparing pairs of protein sequences using a network matching algorithm. The fragments library prediction method (FRAGlib) is also available as the separate http server at [22]. The software is freely available to academics. Contact Dariusz Plewczynski darman@bioinfo.pl or Adam Godzik adam@burnham.org for information on obtaining the local copy of a software.

## Authors' contributions

DP designed, implemented, and evaluated the FRAGlib program. The benchmark dataset and programme for aligning two short sequence profiles were provided by LJ. The integration of FRAGlib predictions within SEA network alignment software together with benchmark evaluation of the SEA method was done by YY. AG was responsible for the overall project coordination. All authors have read and approved the final manuscript.

## Figures and Tables

**Table 1:** General performance of classical methods for building alignments together with segment alignment algorithm incorporating different local structure diversities.

			CE	SEA_T_	**SEA_F_**	SEA_I_	SEA_loc_	BLAST	ALIGN	FFAS
Family (409 pairs)	shift	average		0.61	**0.62**	0.56	0.49	0.44	0.48	0.49
		>0.9		73	**84**	69	47	51	60	43
		>0.7		207	**231**	199	152	146	165	161
		>0.5		282	**277**	260	215	197	228	227
	RMS	≤ 3.0	257	95	**82**	82	63	77	54	40
		≤ 5.0	397	237	**204**	184	147	157	138	118
		≤ 8.0	408	294	**269**	248	231	196	206	194
	all		409	345	**409**	404	366	232	372	409
	len		1	0.84	**1.14**	1.08	0.87	0.56	0.99	1.18

Family-level benchmark for SEA algorithm using FRAGlib's prediction of LSSs (SEA_F_) is compared with SEA_I _(SEA algorithm using I-sites library), SEA_T_, SEA_loc _(local single predicted structures), and other classical tools: CE, BLAST, ALIGN and FFAS. The 'average' is the shift score averaged over all the alignments of the whole subset. The numbers of protein pairs with a shift score or RMSD larger than a certain cut-off value in the subset are listed in columns for each program. The counting based on RMSD requires the length of the alignment to be longer than half of its corresponding structural alignment. The 'all' stands for all the alignments with alignment length no shorter than half of the structural alignments. We use the CE for building reference alignments for shift score calculation, as an example of purely structural alignment tool. The 'len' stands for the average alignment length (predicted aligned position / aligned position in reference alignment from CE). We can see that our method provides very long alignments with relatively good overall score. The difference in the values between SEA_T _and SEA_F _is explained by different lengths of these alignments.
